# The Army Medical Museum and Library

**Published:** 1882-12

**Authors:** 


### THE ARMY MEDICAL MUSEUM AND LIBRARY.

There is every reason to fear that an effort will be made at the next session of Congress to merge these valuable collections into the general congressional library. This of course will destroy their identity, and remove them from the immediate patronage and protection of the medical service of the army and the profession at large. There is no collection in the world comparable with the Museum in some important particulars, and both the Museum and the Library are the special pride of American medicine. In the last report of Surgeon-General C. H. Crane, he recommends that a fire-proof building be provided for these collections, since the present building is inadequate and unsafe. We urge upon every physician who may read this item to use his influence, through his medical society and through individual effort, to induce his representatives in both houses of Congress to give their aid to carrying out the recommendation of the Surgeon-General. Every physician should unite in a concerted effort in this direction, and success will surely follow.—*Louisville Med. News.*

[This subject is worthy the active consideration of all interested in the science of medicine.—Ed.]

